# Artificial Intelligence Predicts Failure of Nonoperative Treatment in Degenerative Posterosuperior Rotator Cuff Tears

**DOI:** 10.1002/ars2.70124

**Published:** 2026-10-20

**Authors:** Santiago Gabardo, Natalia Martínez‐Catalán, José María Belmonte Martínez, Gregorio Bernabé García, José Manuel García Carrasco, Cristina Delgado, María Valencia‐Mora, Gonzalo Luengo‐Alonso, Emilio Calvo

**Affiliations:** ^1^ Shoulder and Elbow Reconstructive Surgery Unit, Department of Orthopedic Surgery and Traumatology, Hospital Universitario Fundación Jiménez Díaz Universidad Autónoma Madrid Spain; ^2^ Computer Engineering Department University of Murcia Murcia Spain

## Abstract

**Purpose:**

To develop an artificial intelligence‐based algorithm to predict success of nonoperative treatment in degenerative posterosuperior tears and to identify the most influential features.

**Methods:**

Patients with degenerative rotator cuff tears initially treated nonoperatively were enrolled prospectively from January 2022 to November 2024. Exclusion criteria included age >75 years; <24 months of follow‐up; massive, traumatic, and partial tears; arthritic changes (Hamada >2); and severe fatty degeneration (Goutallier >2). All patients followed a standardized physiotherapy protocol and could choose surgery if symptoms persisted; failure was defined as opting for surgery. Collected variables included demographics, comorbidities (diabetes, dyslipidemia, hypertension, hypothyroidism, and smoking), magnetic resonance imaging tear characteristics (size; International Society of Arthroscopy, Knee Surgery, and Orthopaedic Sports Medicine classification; associated cuff involvement; and biceps pathology), corticosteroid injection history, and functional scores (American Shoulder and Elbow Surgeons score, Subjective Shoulder Value, and the visual analog scale). First, a standard statistical analysis between groups was performed. Later, the dataset was split into a train (80%) and test (20%), and a random forest algorithm model was developed. Finally, SHapley Additive exPlanations values were computed to assess feature importance.

**Results:**

Of the 412 patients enrolled, 347 patients met the inclusion criteria. After 24 months, 66 (19%) patients opted for surgery. Mean follow‐up was 30.4 months (range, 24‐32). Compared with the successful nonoperative treatment group, patients who failed treatment were younger (58.5 vs 64.6 years, *P* = .001) and had higher body mass index (28.8 vs 27.2, *P* = .016), greater subscapularis involvement (13.5% vs 3%, *P* = .042), higher visual analog scale scores (7.7 vs 7.2, *P* = .018), lower Subjective Shoulder Value (30.9 vs 38.9, *P* = .003), and lower American Shoulder and Elbow Surgeons scores (40.6 vs 47.1, *P* = .001). The model achieved an area under the curve of 84%, predicting success rate of the nonoperative treatment with 94% sensitivity, 62% specificity, 89% positive predictive value, and 77% negative predictive value. The most influential variables for the model were age, tear size, and pretreatment functional scores.

**Conclusions:**

The artificial intelligence‐based algorithm accurately predicted nonoperative treatment outcomes in degenerative posterosuperior rotator cuff tears (area under the curve 0.84, sensitivity 94%). Age, tear size, and function were the main predictors.

**Level of Evidence:**

Level IV, prognostic retrospective case series.

## Graphical abstract

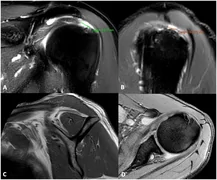

Rotator cuff tears are highly prevalent and represent the leading cause of shoulder pain.^1^, ^2^ They are commonly linked to the natural aging process and repetitive shoulder use.^3^, ^4^, ^5^ Furthermore, with increasing life expectancy and activity levels, the number of rotator cuff repairs has risen sharply, adding clinical and financial pressure on health care systems.^6^, ^7^, ^8^

There is consensus that traumatic tears in young patients should be treated surgically,^9^, ^10^, ^11^, ^12^ whereas elderly patients with comorbidities, massive tears, fatty atrophy, or arthropathy should maximize nonoperative options.^6^ However, for middle‐aged patients with chronic degenerative tears of intermediate characteristics, the most frequent presentation, the optimal approach, remains unclear.^1^, ^4^, ^5^, ^13^

Both nonoperative and surgical treatments aim to reduce pain and improve function, and there is evidence supporting both strategies.^6^, ^11^, ^14^, ^15^ Although surgical treatment for rotator cuff tears has shown favorable outcomes with advances in surgical techniques and postoperative management, nonsurgical treatment remains the initial approach for many patients, particularly those with partial tears, comorbidities, lower functional demands, or reluctance toward surgery.^15^, ^16^ Current practice for nontraumatic, nonmassive degenerative full‐thickness tears usually begins with nonoperative management, including pain control, physiotherapy, lifestyle modification, and corticosteroid injections.^15^, ^16^ Nearly 75% of these patients achieve satisfactory outcomes with this approach.^2^, ^3^, ^13^, ^17^, ^18^, ^19^, ^20^ If symptoms persist after 3 to 6 months, surgery should be considered.^3^, ^21^, ^22^, ^23^, ^24^ In contrast, several studies advocate for prompt surgery, asserting that it presents superior outcomes compared with nonoperative treatment.^25^, ^26^ Although these studies have small sample sizes and show modest differences, their rationale is that nonsurgical management may allow tear size and fatty degeneration progression, whereas repairing smaller tears yields better outcomes.^3^, ^18^, ^20^, ^27^ However, surgery is not without complications, particularly failed healing, which could reach 30% to 40%.^11^, ^28^, ^29^, ^30^

Risk factors for tear development, progression, and repair failure could be divided into patient‐related (age, smoking, diabetes, hypercholesterolemia, and obesity) and tear‐related variables (size, retraction, fatty infiltration, and biceps pathology).^5^, ^18^, ^21^, ^31^, ^32^ However, predicting which patients will benefit from nonoperative treatment remains difficult. Currently, decisions rely on tear characteristics, pain, and functional limitation, as well as surgeon's experience and patient expectations, weighing the risk of tear progression against the potential surgery complications. Given the multifactorial nature of this decision‐making process, tools integrating multiple clinical variables may improve patient selection and individualized treatment.

Artificial intelligence (AI) develops systems capable of learning or decision‐making. Machine learning (ML), a subset of AI, allows systems to learn from data, generating predictions without programming.^33^ ML models can process numerous variables simultaneously, producing accurate and reproducible results. Studies have shown its value in improving diagnostic accuracy and predicting surgical outcomes and complications.^34^, ^35^, ^36^ In rotator cuff management, ML may help identify patients most likely to benefit from early surgery.

The purposes of this study were to develop an AI‐based algorithm to predict the success of nonoperative treatment in degenerative posterosuperior tears and to identify the most influential features. Our hypothesis was that it was feasible to build an accurate and reliable model to support clinical decision‐making.

## METHODS

After this study was approved by the Institutional Review Board of Hospital Universitario Fundación Jiménez Díaz (approval number: EO068‐22_FJD), a prospective database was searched for consecutive patients diagnosed with atraumatic posterosuperior rotator cuff tears between January 2022 and November 2024. All were clinically evaluated by 1 of 6 board‐certified shoulder surgeons at a single institution. At the initial consultation, symptomatic patients presenting with a magnetic resonance imaging (MRI) diagnosis of a degenerative posterosuperior rotator cuff tear were extensively informed about their condition and the available treatment option details, including potential advantages and limitations of nonoperative and surgical treatments. Those who opted for nonsurgical management and met inclusion criteria were enrolled. At this point, demographic, imaging, and functional data were collected. Follow‐up visits were scheduled at 3, 6, 12, and 24 months. Patients could switch to surgery anytime if unsatisfied with progress. Exclusion criteria included patients >75 years old; those with massive, traumatic, or partial tears, substantial fatty atrophy (Goutallier > 2), cuff arthropathy (Hamada > 2), previous shoulder surgeries, instability, inflammatory arthritis, infection, or nerve injury; and those who declined to initiate nonoperative treatment. Recruitment followed equity, diversity, and inclusion principles, without discrimination by sex, ethnicity, or socioeconomic status.

All patients followed the same standardized, unsupervised physiotherapy protocol. Patients received an exercise sheet focused on strengthening the rotator cuff, anterior deltoid, and periscapular musculature, which was explained and shown during the initial consultation. Patients were then referred to their local physiotherapist for follow‐up. Corticosteroid injections were offered to patients with persistent pain as an adjunct to physiotherapy, with the aim of improving pain control and facilitating adherence to the rehabilitation program; injections were administered only to patients who agreed to receive them. Nonoperative treatment failure was defined as conversion to surgery at the patient's request at any time of the follow‐up. Variables were divided into patient demographics and clinical history, tear characteristics, and functional scores. The demographics included age, sex, body mass index (BMI), dominance, occupation (manual or nonmanual), smoking, diabetes, hypercholesterolemia, hypertension, hypothyroidism, and history of contralateral rotator cuff repair and prior corticosteroid injections.

MRI measurements defined tear characteristics. The tear size was measured in millimeters in both the coronal and sagittal views using specific software specifically designed for clinical imaging analysis (Carestream Vue PACS, Carestream Health, Rochester, NY) by S.G. (Figure 1).^37^ Tears were classified based on their size using the International Society of Arthroscopy, Knee Surgery, and Orthopaedic Sports Medicine classification^38^ (C1 < 1 cm, C2 < 2 cm, C3 = 2‐4 cm, and C4 = massive tears). There were no C4 tears because patients with massive tears were excluded. In the sagittal plane, the tendon was divided into 3 sections (anterior, central, and posterior), recording the involved segments. Tears were also divided into smaller or larger than 20 mm, which would correspond to almost the entire tendon.^4^ Fatty infiltration and arthropathy were graded using the MRI Goutallier classification^39^, ^40^ and Hamada classification^41^ on anteroposterior radiograph. Patients with Goutallier or Hamada >3 were excluded because of poor surgical prognosis.^21^, ^30^ Concomitant subscapularis tears were classified per Lafosse,^42^ as recommended by the International Society of Arthroscopy, Knee Surgery, and Orthopaedic Sports Medicine.^38^ For functional evaluation, patients were assessed objectively using the American Shoulder and Elbow Surgeons score,^43^ and the Subjective Shoulder Value^44^ was used as patient‐reported outcome measure to measure function pretreatment. Pain was recorded using the visual analog scale and the presence of night pain. The study adhered to Transparent Reporting of a multivariable prediction model for Individual Prognosis Or Diagnosis guidelines to improve the results reporting quality and transparency.

**FIGURE 1:**
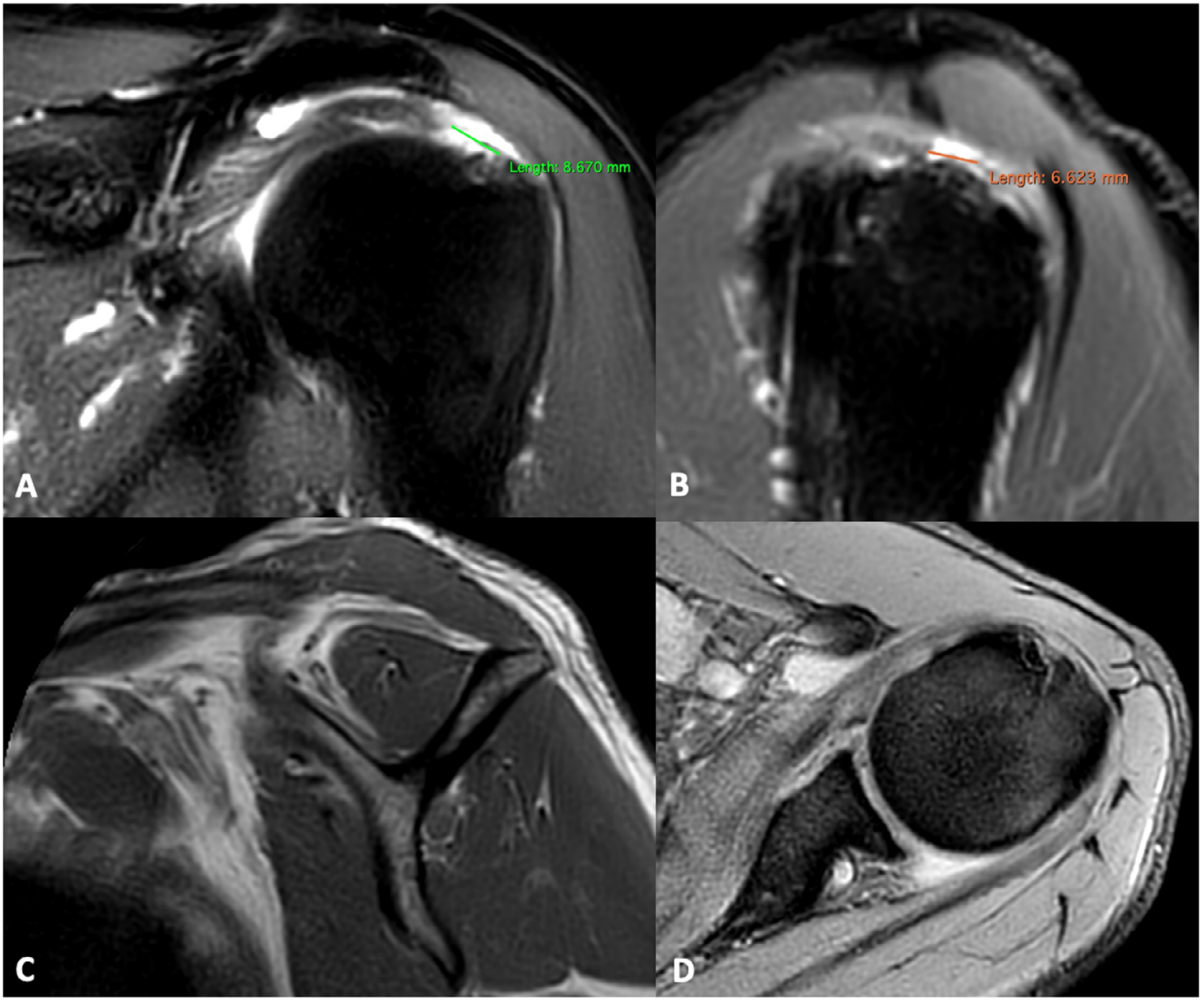
Example of how measurements are taken on an MRI of a left shoulder. (A) Sagittal T2 fat‐sat slice at the level of the supraspinatus tear. (B) Sagittal T2 fat‐sat slice at the level of the tear at the humeral insertion of the supraspinatus tendon. The tear is measured and affects the anterior and middle thirds of the tendon. (C) Sagittal T1 slice at the level of the coracoid process. No fatty atrophy is observed in the supraspinatus (Goutallier grade 0), subscapularis, or infraspinatus. (D) Axial T1 slice showing the integrity of the infraspinatus and subscapularis tendons, with no pathology of the long head of the biceps tendon. (MRI, magnetic resonance imaging.)

### Statistical Analysis and ML Model

Assuming an expected conservative treatment failure rate of 20%, with a 95% confidence level and a 5% margin of error, the minimum required sample size was calculated as 246 patients. However, because multivariable and ML analyses were also planned, and these approaches are sensitive to the number of outcome events, class imbalance, and overall sample size, the recruitment target was increased to 400 patients to account for potential losses to follow‐up and to improve model stability. First, a standard statistical analysis was conducted. Patients were classified into 2 groups: those who remained on nonoperative treatment and those who opted for surgery. Quantitative variables were described with mean and standard deviation and compared using Student's *t* test; qualitative variables were analyzed with *χ* ^2^ or Fisher's exact test. The significance was set at *P* < .05. Logistic regression identified predictors of surgical conversion: univariable analyses for all demographic, clinical, and imaging variables, followed by multivariable regression, including significant or clinically relevant variables. Odds ratios (ORs) and 95% confidence intervals were reported. Analyses were conducted using R (R Foundation for Statistical Computing, Vienna, Austria).

Second, the ML model was subsequently developed. Although k‐fold cross‐validation was tested, no major differences were observed, so a stratified 80/20 train‐test split was used. No missing data were present because of prospective collection. To enhance predictive accuracy, different preprocessing strategies with data‐balancing methods and algorithms were tested. Given the binary outcome and class imbalance, a Balanced Random Forest Classifier was implemented, avoiding resampling bias while maintaining the original data distribution. An evolutionary algorithm performed feature selection, systematically exploring variable combinations to maximize the balanced accuracy. Model development was performed in Python 3.10.0 (Python Software Foundation, Wilmington, DE) using pandas 2.2.3, numpy 2.1.3, and scikit‐learn 1.6.1.

Several methods to evaluate the model's performance were used. Model performance was evaluated using a confusion matrix to derive sensitivity, specificity, positive and negative predictive values, and overall accuracy. The F1‐score and area under the curve (AUC) were also calculated. SHapley Additive exPlanations values were used as an explainability method to quantify the contribution and improve the interpretability of the model. It has been widely used in the literature before in this role.^45^, ^46^

## RESULTS

Of the 412 patients enrolled, 347 patients met the inclusion and exclusion criteria, completed follow‐up, and were analyzed (Figure 2). The mean follow‐up range was 30.4 (24‐32) months. At the end of the follow‐up, 66 (19%) patients had opted for surgery. The average time to surgical intervention for those patients was 7.7 months (standard deviation: 3.3), with a median of 6.7 months from inclusion. The basic characteristics of each group and descriptive analysis are illustrated in Table 1. Significant differences between success and failure groups included age (58.5 vs 64.6 years, *P* = .001), BMI (28.8 vs 27.2, *P* = .016), subscapularis involvement (3% vs 13.5%, *P* = .042), visual analog scale (7.7 vs 7.2, *P* = .018), Subjective Shoulder Value (30.9 vs 38.9, *P* = .003), and American Shoulder and Elbow Surgeons (40.6 vs 47.1, *P* = .001). Tear size was not significantly associated with the outcome, with no differences observed in sagittal (16.5 vs 15.6 mm, *P* = .38) or coronal dimensions (13.5 vs 14.9 mm, *P* = .062). In the multivariable logistic regression analysis (Figure 3), younger age (OR 0.90, [0.86‐0.93, *P* < .001]), higher BMI (OR 1.07, [1.01‐1.14, *P* = .018]), larger tear size in the sagittal plane (OR 1.16, [1.07‐1.26, *P* < .001]), and smaller size in the coronal plane (OR 0.93, [0.88‐0.99, *P* = .023]) were independently associated with an increased likelihood of failing nonoperative treatment. Functional scores showed a trend but did not reach statistical significance.

**FIGURE 2:**
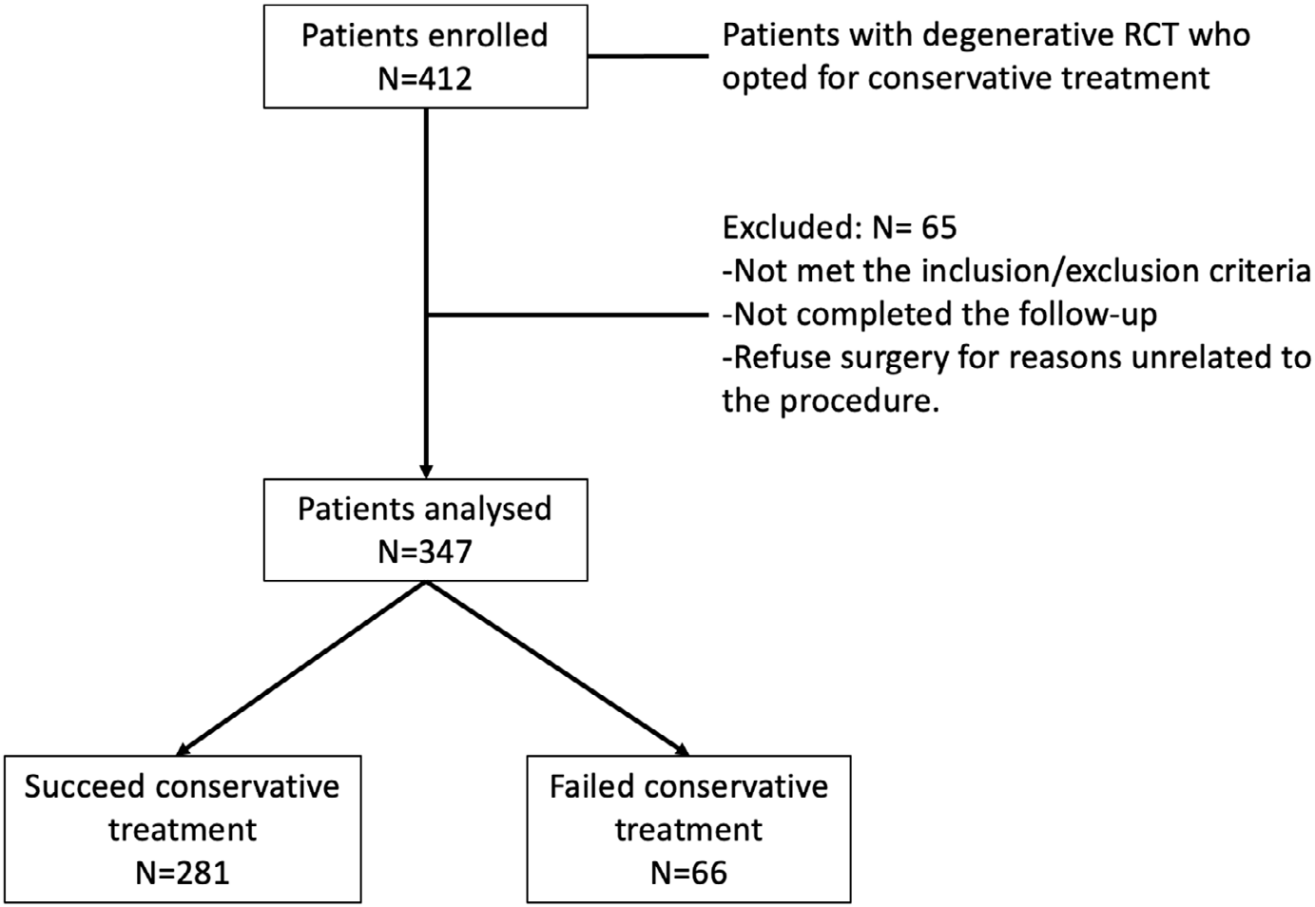
Flowchart of patient selection and follow‐up. Of the 412 patients initially recruited with degenerative posterosuperior tears, 65 were excluded. A total of 347 patients were included in the final analysis, with 66 (19%) undergoing surgery after failure of nonoperative treatment and 281 (81%) remaining on nonoperative management during the follow‐up. (N, number; RCT, rotator cuff tears.)

**TABLE 1:** The Basic Characteristics of Each Group and the Descriptive Analysis

	**Total N = 347**	**Success N = 281**	**Failure N = 66**	***P***	**OR (95% CI)**
Age, *x̅* (SD)	63.5 (7.6)	64.6 (7.6)	58.5 (8.1)	**.001**	**0.90 (0.87, 0.94)**
Women, N (%)	230 (66%)	185 (65.8%)	47 (71.2%)	.404	0.82 (0.45, 1.46)
Hand dominance, N (%)	234 (67%)	186 (66.2%)	50 (75.8%)	.134	1.50 (0.83, 2.80)
Manual worker, N (%)	169 (48.9%)	132 (47%)	39 (59.1%)	.076	1.55 (0.91, 2.69)
BMI, *x̅* (SD)	27.5 (4.8)	27.2 (4.9)	28.8 (4.8)	**.016**	**1.07 (1.01, 112)**
ISAKOS classification					
C1, N (%)	171 (49.1%)	140 (49.8%)	31 (47%)	.677	‐
C2, N (%)	130 (37.6%)	99 (35.2%)	31 (47%)	.076	1.41 (0.81, 2.48)
C3, N (%)	46 (13.2%)	42 (14.9%)	4 (6.1%)	.055	0.43 (0.12, 1.16)
SizeSag, *x̅* (SD)	16.3 (7.6)	16.5 (7.6)	15.6 (6.6)	.38	0.98 (0.95, 1.02)
SizeCor, *x̅* (SD)	13.8 (5.4)	13.5 (5.3)	14.9 (4.5)	.062	1.05 (1.00, 1.10)
Complete (>20 mm), N (%)	105 (30.3%)	86 (30.6%)	19 (28.8%)	.772	0.92 (0.50, 163)
Tear localization					
Anterior third, N (%)	244 (70%)	194 (69%)	50 (75.8%)	.282	1.40 (0.77, 2.67)
Central third, N (%)	190 (54.7%)	158 (56.2%)	33 (50%)	.36	0.75 (0.44, 1.30)
Posterior third, N (%)	92 (26.5%)	76 (27%)	16 (24.2%)	.642	0.86 (0.45, 1.30)
Infraspinatus involvement, N (%)	162 (46.7%)	134 (47.7%)	28 (42.4%)	.441	0.81 (0.47, 1.38)
Subscapularis involvement, N (%)	242 (69.7%)	200 (71.2%)	42 (63.6%)	.231	0.71 (0.41, 1.26)
Lafosse I, N (%)	201 (57.9%)	162 (57.6%)	39 (59.1%)	.831	0.33 (0.08, 0.97)
Lafosse II, N (%)	41 (12.4%)	38 (13.5%)	3 (4.5%)	**.042**	1.23 (0.69, 2.17)
LHBT					
Subluxed, N (%)	131 (37.5%)	104 (37%)	27 (40.9%)	.557	1.49 (0.52, 5.41)
Partial tear, N (%)	65 (18.7%)	50 (17.8%)	15 (22.7%)	.3553	1.73 (0.55, 6.56)
Normal, N (%)	105 (30.3%)	85 (30.2%)	20 (30.3%)	.993	1.35 (0.46, 4.99)
Dislocated, N (%)	19 (5.5%)	19 (6.8%)	0 (0%)	**.029**	0.01 (0.00, 2.68)
Absent, N (%)	27 (7.8%)	23 (8.2%)	4 (6.1%)	.562	‐
Any injury, N (%)	242 (69.7%)	196 (69.7%)	46 (69.7%)	.993	1.00 (0.56, 1.82)
Goutallier					
0, N (%)	79 (22.8%)	65 (23.1%)	14 (21.2%)	.738	‐
I, N (%)	200 (57.6%)	157 (55.9%)	43 (65.1%)	.169	‐
II, N (%)	68 (19.5%)	59 (21%)	9 (13.6%)	.175	0.88 (0.58, 1.33)
Previous corticosteroid injections, N (%)	93 (26.8%)	75 (26.7%)	18 (27.3%)	.923	1.13 (0.55, 1.86)
Total corticosteroid injections, *x̅* (SD)	0.3 (0.58)	0.32 (0.6)	0.33 (0.6)	.994	1.04 (0.64, 1.61)
Hamada					
0, N (%)	119 (34.3%)	97 (34.5%)	22 (33.3%)	.855	‐
I, N (%)	160 (46.1%)	126 (44.8%)	31 (47%)	.754	‐
II, N (%)	69 (19.9%)	58 (20.6%)	10 (15.1%)	.173	0.92 (0.63, 1.33)
Smoking, N (%)	74 (21.3%)	64 (22.8%)	10 (15.1%)	.174	0.61 (0.28, 1.21)
Diabetes, N (%)	38 (10.9%)	33 (11.7%)	5 (7.6%)	.329	0.62 (0.20, 1.51)
Dyslipidemia, N (%)	121 (34.9%)	99 (35.2%)	22 (33.3%)	.771	0.92 (0.51, 1.61)
High blood pressure, N (%)	115 (33.1%)	95 (33.8%)	20 (30.3%)	.586	0.85 (0.47, 1.50)
Hypothyroidism, N (%)	42 (12.1%)	28 (10%)	8 (12.1%)	.549	1.25 (0.51, 2.76)
Contralateral side repair, N (%)	26 (7.5%)	18 (6.4%)	8 (12.1%)	.112	2.02 (0.79, 4.72)
VAS, *x̅* (SD)	7.3 (1.7)	7.2 (1.8)	7.7 (1.7)	**.018**	**1.23 (1.04, 1.47)**
Night pain, N (%)	263 (75.8%)	209 (74.3%)	54 (81.8%)	.272	1.55 (0.81, 3.19)
SSV, *x̅* (SD)	37.3 (19.5)	38.8 (19.6)	30.9 (20)	**.003**	**0.98 (0.96, 0.99)**
ASES, *x̅* (SD)	46.4 (16.2)	47.1 (16.1)	40.6 (14.6)	**.001**	**0.97 (0.95, 0.99)**

*Note*: The *P* values that have been statistically significant are in bold.

ASES, American Shoulder and Elbow Surgeons Score; BMI, body mass index; CI, confidence interval; ISAKOS, International Society of Arthroscopy, Knee Surgery, and Orthopaedic Sports Medicine; LHBT, long head of the biceps tendon; N, number; OR, odds ratio; SD, standard deviation; SizeCor, tear size in coronal plane in millimeters; SizeSag, tear size in sagittal plane in millimeters; SSV, Subjective Shoulder Value; *x̅*, mean; VAS, visual analog scale.

**FIGURE 3:**
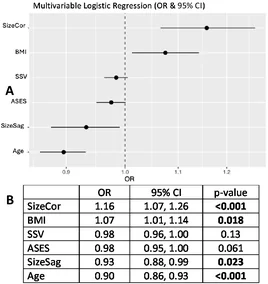
Multivariable logistic regression analysis results (A) and forest plot (B). The *P* values that have been statistically significant are in bold. (ASES, American Shoulder and Elbow Surgeons Score; BMI, body mass index; CI, confidence intervals; OR, odds ratios; SizeCor, tear size in coronal plane in millimeters; SizeSag, tear size in sagittal plane in millimeters; SSV, Subjective Shoulder Value.)

Initial models trained on the unbalanced dataset showed poor accuracy. When data‐balancing techniques were applied, performance improved, yielding AUC values above 0.70. The best results were obtained after applying the evolutionary algorithm to the balanced dataset, which selected 13 variables for the final model. The model's performance on the test split is illustrated in the confusion matrix (Figure 4). The model achieved a sensitivity of 94%, a specificity of 63%, a positive predictive value of 89%, a negative predictive value of 77%, a global accuracy of 83%, and an F1‐score of 91.7%. The AUC was 0.84 (0.689‐0.963) (Figure 5).

**FIGURE 4:**
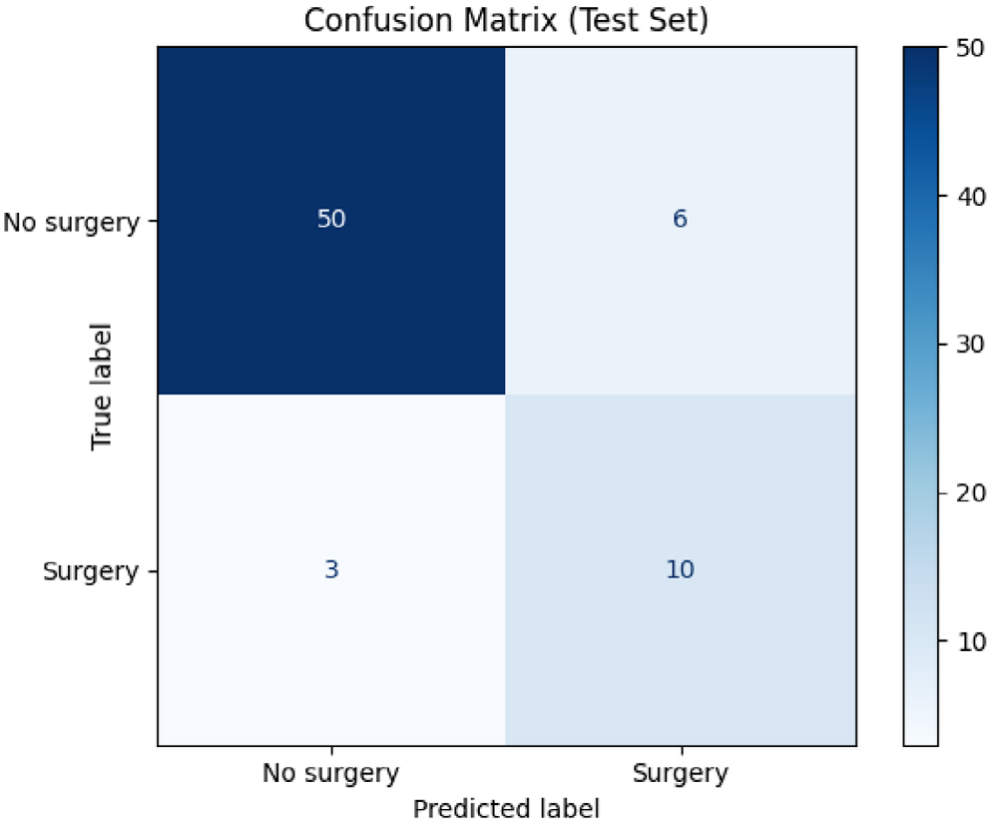
Confusion matrix to represent the results of the algorithm generated on the test dataset split. On the horizontal axis, the predicted labels are shown, which represent the classifications made by the model based on the data samples, whereas the vertical axis displays the true labels, referring to the actual classification of the collected data. The upper‐left box corresponds to the true positives, the upper‐right box to the false positives, the lower‐left box to the false negatives, and the lower‐right box to the true negatives.

**FIGURE 5:**
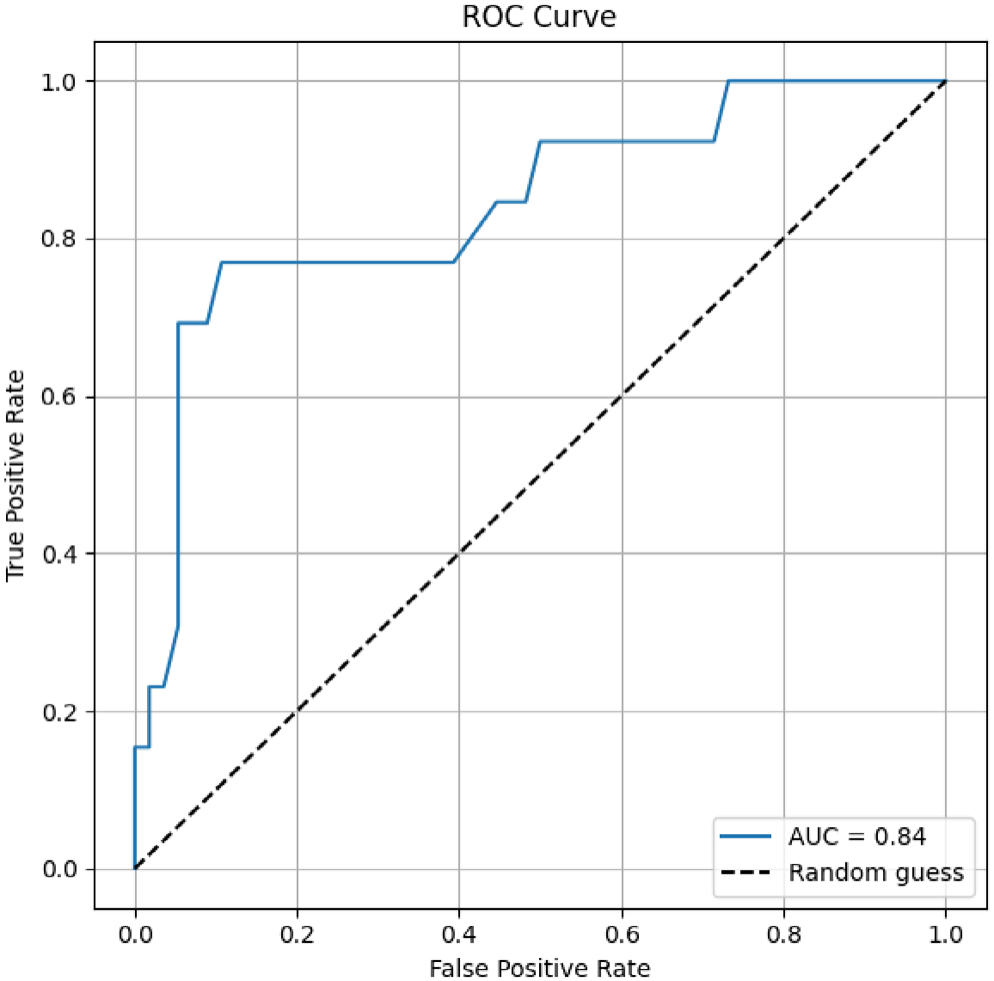
ROC curve of the predictive model. The curve illustrates the diagnostic performance of the machine learning algorithm in predicting successful nonoperative treatment of rotator cuff tears. The AUC is 0.84, indicating good discriminative ability. (AUC, area under the curve; ROC, receiver operating characteristic.)

Regarding the SHapley Additive exPlanations analysis of the variables, all variables included in the model were found to be significant for its predictions (Figure 6). Nonetheless, the most important were age, tear size in both planes, BMI, and preoperative functional scales. However, to achieve the best performance, the model selected 13 variables (age; BMI; International Society of Arthroscopy, Knee Surgery, and Orthopaedic Sports Medicine classification; tear size in both coronal and sagital planes; tear location; tear larger than 20 mm; involvement of the infraspinatus; fatty atrophy; previous infiltrations; Hamada classification; diabetes; and smoking), and functional scales were not included.

**FIGURE 6:**
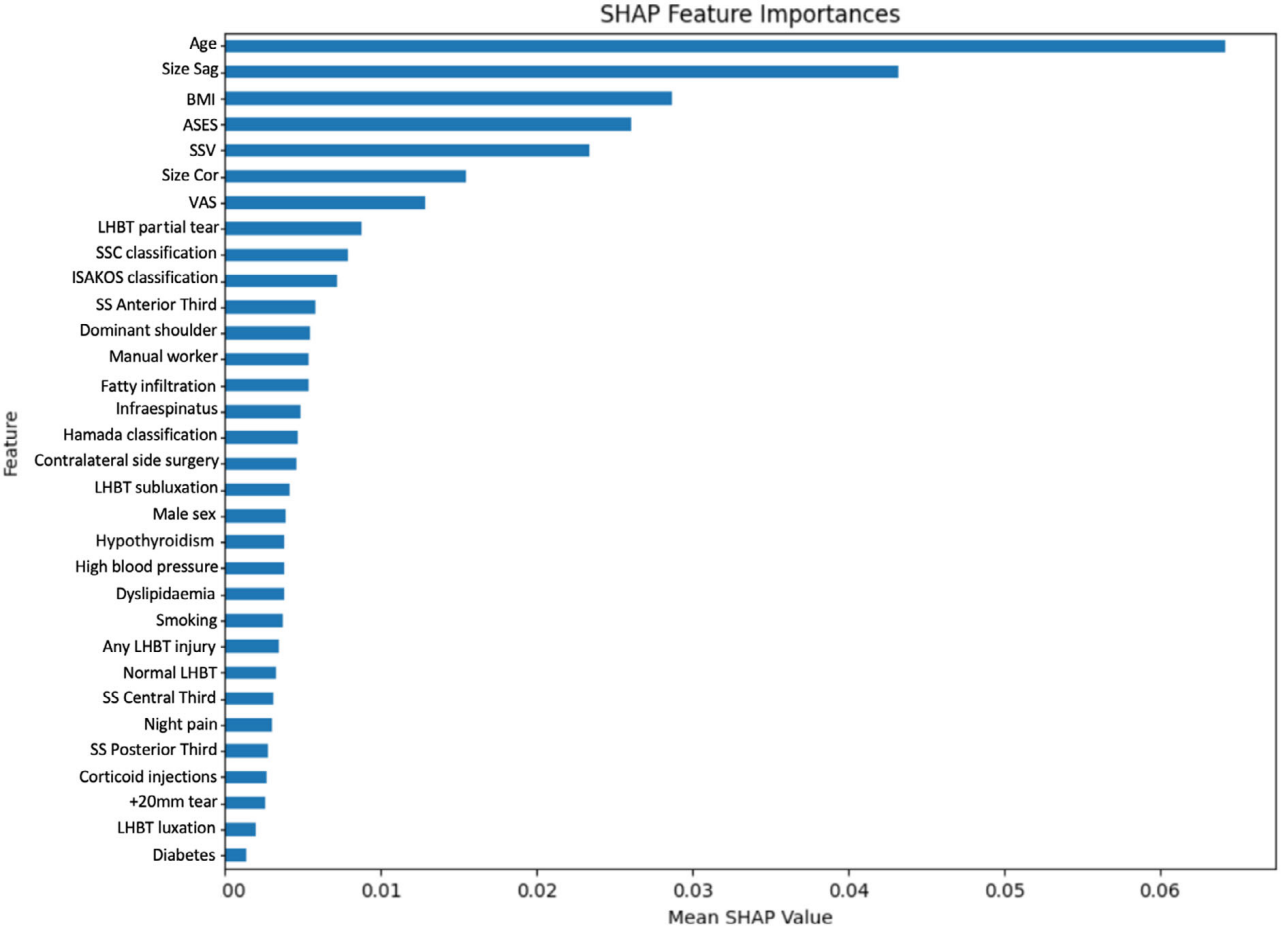
Diagram of the importance of each variable for the model according to the SHAP technique. (ASES, American Shoulder and Elbow Surgeons Score; BMI, body mass index; ISAKOS, International Society of Arthroscopy, Knee Surgery, and Orthopaedic Sports Medicine; LHBT, long head of the biceps tendon; SHAP, SHapley Additive exPlanations; SizeCor, tear size in coronal plane in millimeters; SizeSag, tear size in sagittal plane in millimeters; SS, supraspinatus; SSC, subscapularis; SSV, Subjective Shoulder Value; VAS, visual analog scale.)

## DISCUSSION

The main finding of this study is that an ML model capable of predicting, with acceptable accuracy, the success of nonoperative treatment for degenerative rotator cuff tears can be developed. Additionally, it was found that the features that influence this decision the most were age, tear size, BMI, and preoperative functional scales.

Management of rotator cuff tears remains controversial, balancing surgical risks against progression under nonoperative treatment because both can yield good results. Several studies report successful results with nonsurgical management in up to 75% of patients,^13^, ^17^, ^18^, ^19^, ^20^ consistent with our findings (81%), and these benefits appear to be sustained over time.^47^ Nonetheless, Moosmayer et al. prospectively followed 105 patients at 2, 5, and 10 years and found that those who underwent surgery after failed nonoperative treatment had worse outcomes than those operated on initially.^48^, ^49^, ^50^ Therefore, identifying patients likely to fail nonoperative management could allow earlier surgery, improving outcomes and optimizing resources. AI could be decisive in this task.

AI has opened the door to countless discoveries, from new antibiotics to optimized cancer treatments and improved operating room efficiency.^51^, ^52^ AI‐based tools are expected to become increasingly common in clinical practice. In orthopaedics, progress has been most notable in imaging diagnostics through deep learning, which applies neural networks to large datasets.^53^ These models detect fractures on plain radiographs with accuracy comparable to expert radiologists, improving accuracy and interpretation time.^54^, ^55^, ^56^ Beyond diagnostics, predictive models have been developed to estimate complications, length of stay, and outcomes after procedures such as spine fusions and joint replacements.^34^, ^35^, ^57^, ^58^

Our model, with an AUC of 0.84% and 94% sensitivity, achieved strong predictive performance.^53^ It reliably identifies patients likely to succeed with nonoperative treatment, supporting clinical decision‐making. Previous studies using ML in rotator cuff repair have shown heterogeneous results. Some achieved only modest accuracy in predicting postoperative functional outcomes.^59^, ^60^ Potty et al.^45^ successfully identified patients likely to experience clinical improvement after surgery, whereas other models focusing on retear prediction showed particularly strong performance.^61^, ^62^ Beyond overall performance metrics, model interpretability is essential for clinical adoption. Figure 7 illustrates how the model integrates individual patient characteristics to generate a personalized prediction, showing the relative contribution of each variable to the final outcome and exemplifying how the algorithm could be used in clinical practice to support transparent, patient‐specific decision‐making.

**FIGURE 7:**
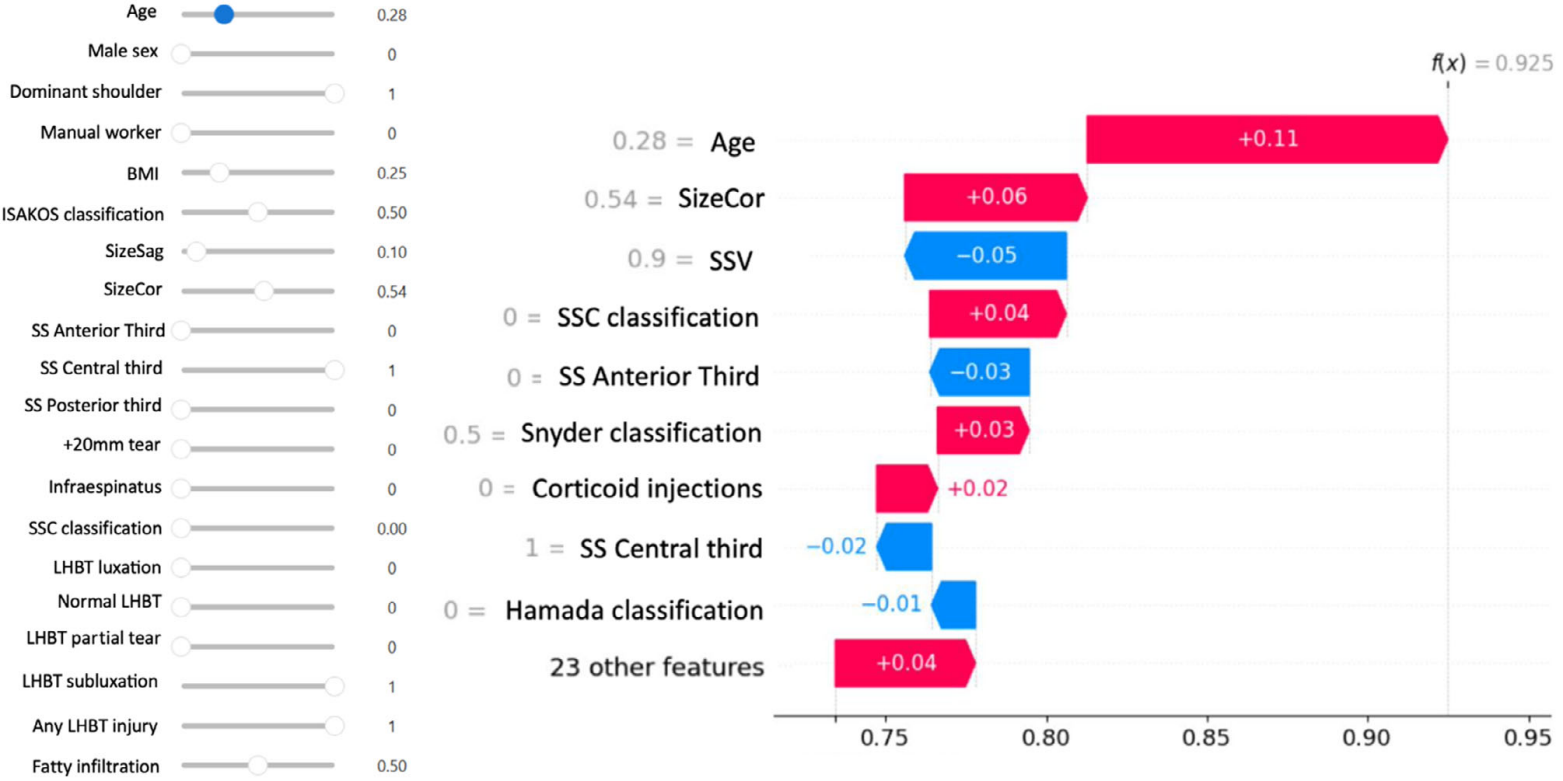
SHAP waterfall plot illustrating an individual prediction of the machine learning model. The diagram shows how each clinical, functional, and imaging variable contributes to the final predicted probability of failure of nonoperative treatment for a single patient. Features in red increase the predicted probability, whereas features in blue decrease it. The cumulative effect of all variables shifts the prediction from the baseline value to the final output, providing an interpretable explanation of the model's decision. In this example, the patient has a 92.5% chance of failing nonoperative treatment. (BMI, body mass index; ISAKOS, International Society of Arthroscopy, Knee Surgery, and Orthopaedic Sports Medicine; LHBT, long head of the biceps tendon; SHAP, SHapley Additive exPlanations; SizeCor, tear size in coronal plane in millimeters; SizeSag, tear size in sagittal plane in millimeters; SS, supraspinatus; SSC, subscapularis; SSV, Subjective Shoulder Value.)

Younger age is the main risk factor for nonoperative treatment failure in rotator cuff tears.^1^, ^18^, ^29^ In our study, it was not only significant in the multivariable analysis but also the most influential predictor for the model, with younger patients more likely to undergo surgery. Higher BMI, previously linked to poor repair outcomes,^32^ also showed high predictive importance for nonoperative treatment failure. As shown in Figure 6, SHapley Additive exPlanations analysis assigned little importance to comorbidities such as dyslipidemia, hypothyroidism, diabetes, or smoking. However, diabetes and smoking were retained by the model to enhance accuracy, consistent with their known link to tear progression.^3^, ^15^, ^20^ A similar pattern was observed for corticosteroid injections: although no differences emerged in the descriptive analysis, the model identified them as useful predictors, aligning with literature supporting their role in pain reduction and adherence to physiotherapy.^19^, ^63^ Conversely, factors such as sex or prior contralateral cuff repair were not associated with prognosis in either analysis.

Among tear‐related factors, the size in both planes was a major determinant of nonoperative treatment success. Wider anteroposterior tears with less coronal retraction carried higher failure risk, likely because larger tears cause greater biomechanical disruption and progress faster, whereas retraction alone is less critical.^2^, ^18^, ^37^ Fatty atrophy and arthropathy, recognized poor prognostic factors in the literature,^21^, ^30^ were also identified by the model, reflecting consistency of the AI model with current evidence. The number of tendons involved was relevant because multitendon tears cause greater dysfunction,^28^ and both analyses confirmed the predictive value of combined subscapularis and infraspinatus involvement. In contrast, long head of biceps pathology, often associated with cuff disease,^28^ was not significant.

The absence of functional status and pain among selected variables, despite their statistical significance, illustrates the “black box” phenomenon.^61^ This refers to the difficulty in understanding why the model makes certain decisions. Adding more variables does not always improve accuracy; often, seemingly relevant data reduce precision. Oeding et al.^46^ reported a similar finding when combining clinical and radiological data to detect subscapularis tears; the algorithm ignored clinical variables because they reduced the accuracy.

Our study has many strengths. Being prospective ensures excellent data quality, crucial for robust ML performance.^53^ The cohort is also highly homogeneous with strict exclusion criteria, only evaluating patients with complete degenerative tears who began nonoperative treatment, all analyzed through MRI imaging. Additionally, the variables identified by the model match those reported as prognostically relevant, supporting its clinical applicability. Finally, this study provides promising results that may guide future research, contributing to the development of predictive tools that assist surgeons in decision‐making and promote patient‐tailored care.

### Limitations

This study is not without limitations. Although the sample size was adequate for conventional statistical analysis, larger cohorts are generally desirable in AI‐based studies to improve model accuracy, robustness, and clinical implementation. Some factors previously identified as potentially relevant, such as muscle strength, occupation, workload, recreational or sports activities, range of motion, psychological factors, and socioeconomic variables, were not collected and therefore could not be incorporated into the analysis.^1^, ^8^, ^64^, ^65^ However, variables previously recognized as clinically relevant in the prognosis of nonsurgical treatment of rotator cuff tears were identified, proving the validity of this model. Although sex was included as an independent variable and was not significantly associated with the main outcomes in any of the analyses performed, sex‐disaggregated analyses were not conducted. This represents a limitation because potential sex‐specific differences may not have been fully explored. Although the 24‐month follow‐up might seem short, Kuhn et al.^17^ found that patients not requiring surgery within 12 months rarely did so later, consistent with our findings that most surgical decisions occurred within the first year. Additionally, physiotherapy adherence and progression were not directly supervised, which may have introduced variability in treatment implementation. Finally, because the algorithm was trained with data from a single institution, external validation is needed.

## CONCLUSIONS

The AI‐based algorithm accurately predicted nonoperative treatment outcomes in degenerative posterosuperior rotator cuff tears (AUC 0.84, sensitivity 94%). Age, tear size, and function were the main predictors.

## DISCLOSURES

The authors (J.M.B.M., G.B.G., J.M.G.C.) declare the following financial interests/personal relationships which may be considered potential competing interests: J.M.B.M., G.B.G., J.M.G.C. report relationships with Ministerio de Ciencia, Innovación y Universidades; Agencia Estatal de Investigación; European Union NextGenerationEU; and Plan de Recuperación, Transformación y Resiliencia. All other authors (S.G., N.M‐C., C.D., M.V‐M., G.L‐A., E.C.) declare that they have no known competing financial interests or personal relationships that could have appeared to influence the work reported in this article.
